# Diagnostic radiology and its future: what do clinicians need and think?

**DOI:** 10.1007/s00330-023-09897-2

**Published:** 2023-07-12

**Authors:** Thomas C. Kwee, Maan T. Almaghrabi, Robert M. Kwee

**Affiliations:** 1 Department of Radiology, Medical Imaging Center, University Medical Center Groningen, University of Groningen, Hanzeplein 1, P.O. Box 30.001, 9700 RB Groningen, The Netherlands; 2 Department of Radiology, Zuyderland Medical Center, Heerlen/Sittard/Geleen, The Netherlands

**Keywords:** Artificial intelligence, Radiology, Value-based healthcare, Workforce

## Abstract

**Objective:**

To investigate the view of clinicians on diagnostic radiology and its future.

**Methods:**

Corresponding authors who published in the *New England Journal of Medicine* and the *Lancet* between 2010 and 2022 were asked to participate in a survey about diagnostic radiology and its future.

**Results:**

The 331 participating clinicians gave a median score of 9 on a 0–10 point scale to the value of medical imaging in improving patient-relevant outcomes. 40.6%, 15.1%, 18.9%, and 9.5% of clinicians indicated to interpret more than half of radiography, ultrasonography, CT, and MRI examinations completely by themselves, without consulting a radiologist or reading the radiology report. Two hundred eighty-nine clinicians (87.3%) expected an increase in medical imaging utilization in the coming 10 years, whereas 9 clinicians (2.7%) expected a decrease. The need for diagnostic radiologists in the coming 10 years was expected to increase by 162 clinicians (48.9%), to remain stable by 85 clinicians (25.7%), and to decrease by 47 clinicians (14.2%). Two hundred clinicians (60.4%) expected that artificial intelligence (AI) will not make diagnostic radiologists redundant in the coming 10 years, whereas 54 clinicians (16.3%) thought the opposite.

**Conclusion:**

Clinicians who published in the *New England Journal of Medicine* or the *Lancet* attribute high value to medical imaging. They generally need radiologists for cross-sectional imaging interpretation, but for a considerable proportion of radiographs, their service is not required. Most expect medical imaging utilization and the need for diagnostic radiologists to increase in the foreseeable future, and do not expect AI to make radiologists redundant.

**Clinical relevance statement:**

The views of clinicians on radiology and its future may be used to determine how radiology should be practiced and be further developed.

**Key Points:**

*• Clinicians generally regard medical imaging as high-value care and expect to use more medical imaging in the future.*

*• Clinicians mainly need radiologists for cross-sectional imaging interpretation while they interpret a substantial proportion of radiographs completely by themselves.*

*• The majority of clinicians expects that the need for diagnostic radiologists will not decrease (half of them even expect that we need more) and does not believe that AI will replace radiologists.*

**Supplementary information:**

The online version contains supplementary material available at 10.1007/s00330-023-09897-2.

## Introduction

Diagnostic radiology has evolved tremendously over the past decades. Diagnostic accuracy has improved owing to the advent of cross-sectional imaging techniques, imaging volumes have increased, and radiologists are increasingly involved in multidisciplinary clinical decision-making [1–3]. Meanwhile, concepts such as precision medicine and value-based healthcare have been introduced to reform clinical practice [4, 5], and radiology leaders have put forward potential strategies to accommodate them [6, 7]. However, major challenges that still need to be addressed in the present and the foreseeable future are the escalating healthcare costs and the increasing workload of radiologists due to the ever-rising demand for medical imaging services [2, 8–10].

Cuts in healthcare reimbursements for radiological procedures and an unwillingness to train more radiologists conflict with the projected increase in radiology workload. Introducing artificial intelligence (AI) tools in radiology has been proposed as a potential solution to aid efficiency, ease workload, and perhaps even replace radiologists [11–14]. However, widespread implementation of AI has not been realized yet in routine practice. Consequently, policymakers may be unsure about how diagnostic radiology will develop in the future in terms of required investments and workforce. Policymakers may also wonder if medical imaging adds value to healthcare [5], and if it is worthwhile to be invested in. Because of these unresolved issues and tight budgets, investments in radiology are not infrequently stalled.

Radiologists work in close collaboration with clinicians in providing healthcare services to patients. Currently, there is a lack of literature on how clinicians view radiology and its future with respect to required services, whether or not these services add value, and the role of AI in the profession. This information plays a crucial role in how radiology should be practiced and can be prepared for the future.

The purpose of this study was therefore to investigate the view of clinicians on diagnostic radiology and its future.

## Materials and methods

### Study participants

This survey study was approved by the institutional review board of the University Medical Center Groningen. The *New England Journal of Medicine* and the *Lancet* are regarded as the prime journals for publishing studies that have a major impact on clinical practice. All corresponding authors of articles that were published in these two journals between January 2010 and September 2022 were sent an invitation to participate in a survey about the value and future of radiology. The e-mail address of the corresponding author that was provided in each article was used for this purpose. Survey participants without a medical doctor (MD) degree and those with radiology and/or nuclear medicine as medical specialty were excluded. The first invitations for survey participation were sent on 28 September 2022, and reminders were sent on 12 October, 26 October, 16 November, and 30 November 2022.

### Survey

The survey contained 18 closed-ended questions, with 9 questions capturing information on respondents’ basic characteristics (age, gender, type of workplace, country of work, possession of an MD degree, years of experience as an MD, medical specialty qualification, and current number of weekly working days in clinical practice), 5 questions on which diagnostic radiology examinations are requested by the respondents and by whom these are interpreted, and 4 questions on the value of medical imaging utilization and expected future developments (Table 1). Finally, the survey participants were given the opportunity to leave any comments in a free text field. The digital survey was built with Qualtrics Core XM survey software (Qualtrics LLC) and access was provided through a weblink. Participation was on an anonymous basis.

**Table 1:** Survey questions and answer options

No	Question	Answer options
1	How old are you?	< 18, 18–24, 25–34, 35–44, 45–54, 55–64, or > 65 years old
2	What is your gender?	Male, female, or other
3	Where do you currently work or have you most recently worked?*	Academic hospital, non-academic hospital, non-hospital healthcare setting, other
4	In which country do you work or have you most recently worked?*	List of 30 countries, and open text field to indicate another country not listed
5	Are you a medical doctor?	Yes or no
6	How many years of experience do you have as a medical doctor?	< 5, 5–10, or > 10 years
7	Are you a medical specialist?	Yes or no
8	What is your medical specialty?*	List of 38 medical specialties, and open text field to indicate another medical specialty not listed
9	How many days per week do you currently work in clinical practice?	0, 1, 2, 3, 4, 5, 6, or 7
10	What type(s) of diagnostic radiology examinations do you request in clinical practice?*	None, plain radiography, ultrasonography, CT, MRI
11	How many of your plain radiographs do you interpret completely by yourself, without consulting a radiologist or reading his/her report?	None, 1–25%, 26–50%, 51–75%, 76–99%, or 100%
12	How many of your ultrasonography examinations do you perform and interpret completely by yourself, without consulting a radiologist or reading his/her report?	None, 1–25%, 26–50%, 51–75%, 76–99%, or 100%
13	How many of your CT examinations do you interpret completely by yourself, without consulting a radiologist or reading his/her report?	None, 1–25%, 26–50%, 51–75%, 76–99%, or 100%
14	How many of your MRI examinations do you interpret completely by yourself, without consulting a radiologist or reading his/her report?	None, 1–25%, 26–50%, 51–75%, 76–99%, or 100%
15	In your opinion, how much does medical imaging contribute to improving patient-relevant outcomes?	0–10 point linear scale, with 0 indicating nothing and 10 indicating very much
16	Do you expect that medical imaging utilization will increase in the coming 10 years?	Yes, no, or undecided
17	Do you expect that we need more or less diagnostic radiologists in the coming 10 years?	More, neither more nor less, less, or undecided
18	Do you expect that artificial intelligence will make diagnostic radiologists redundant in the coming 10 years?	Yes, no, or undecided

*Multiple answer options possible

### Data analysis

Basic characteristics of the respondents, their opinion on the value of medical imaging, their habits towards requesting and interpreting diagnostic radiology examinations, and expected future developments were descriptively summarized. Multivariate regression analyses were performed to determine the association between clinicians’ dependency on radiologists in interpreting diagnostic radiology examinations (according to questions 11–14 as shown in Table 1) with the type of clinician (academic vs. non-academic hospital employment, medical specialist vs. non-medical specialist status, and surgical specialty vs. non-surgical specialty) and continent of work (Africa, Asia, Australia, Europe, North America, or South America). Note that cardiothoracic surgery, neurosurgery, obstetrics and gynecology, ophthalmology, oral and maxillofacial surgery, orthopedics, otorhinolaryngology, plastic surgery, general, oncologic, pediatric, transplant, and vascular surgery, and urology were predefined as surgical specialties, and all other specialties were regarded as non-surgical specialties, as also indicated in Table 2. Clinicians with both a surgical and a non-surgical specialty were included in the surgical specialty group. Multivariate regression analyses were also performed to determine the associations between the value attributed to medical imaging, projected medical imaging utilization, radiologist workforce requirement, and role of AI, with the clinician’s characteristics (age, gender, academic vs. non-academic hospital employment, continent of work, medical specialist vs. non-medical specialist status, surgical specialty vs. non-surgical specialty, years of clinical experience as MD). *p*-values less than 0.05 were considered statistically significant. Statistical analyses were executed using IBM Statistical Package for the Social Sciences (SPSS) version 23. The comments provided by the respondents in the free text field at the end of the survey were jointly analyzed by the authors (T.C.K., M.T.A., and R.M.K.) to identify the most common topics that were put forward.

**Table 2:** Characteristics of 331 clinicians

Variable	Category	Count	Percentage
Age	< 18 years	1	0.3%
	18–24 years	2	0.6%
	25–34 years	23	6.9%
	35–44 years	60	18.1%
	45–54 years	77	23.3%
	55–64 years	89	26.9%
	> 65 years	79	23.9%
Gender	Male	246	74.3%
	Female	83	25.1%
	Other	2	0.60%
Type of workplace	Academic hospital	271	81.9%
	Non-academic hospital	22	6.6%
	Non-hospital healthcare setting	11	3.3%
	Other workplace	18	5.4%
	Combination of different workplaces	9	2.7%
Country of work	Argentina	1	0.3%
	Australia	11	3.3%
	Austria	1	0.3%
	Belgium	7	2.1%
	Brazil	4	1.2%
	Canada	23	6.9%
	China	2	0.6%
	Colombia	2	0.6%
	Costa Rica	1	0.3%
	Cyprus	1	0.3%
	Denmark	1	0.3%
	Finland	1	0.3%
	France	11	3.3%
	Germany	8	2.4%
	Greece	3	0.9%
	India	13	3.9%
	Indonesia	1	0.3%
	Iran	1	0.3%
	Ireland	1	0.3%
	Israel	4	1.2%
	Italy	16	4.8%
	Japan	2	0.6%
	Kazakhstan	1	0.3%
	Malaysia	3	0.9%
	Mexico	2	0.6%
	New Zealand	1	0.3%
	Norway	4	1.2%
	Palestine	2	0.6%
	Peru	2	0.6%
	Romania	2	0.6%
	South Africa	5	1.5%
	Spain	9	2.7%
	Sweden	8	2.4%
	Switzerland	8	2.4%
	Tanzania	2	0.6%
	Thailand	1	0.3%
	The Netherlands	32	9.7%
	Turkey	4	1.2%
	Uganda	3	0.9%
	UK	56	16.9%
	USA	64	19.3%
	Zimbabwe	1	0.3%
	Multiple countries	6	1.8%
Medical specialist	Yes	307	92.7%
	No	24	7.3%
Medical specialty	Allergy and immunology^d^	1	0.3%
	Anesthesiology^d^	6	2.0%
	Cardiology^d^	15	4.9%
	Cardiothoracic surgery^c^	1	0.3%
	Chemical pathology and metabolic medicine^d^	1	0.3%
	Clinical genetics^d^	1	0.3%
	Clinical pharmacology^d^	1	0.3%
	Dermatology^d^	3	1.0%
	Emergency medicine^d^	6	2.0%
	Endocrinology^d^	11	3.6%
	Family medicine^d^	4	1.3%
	Gastroenterology^d^	8	2.6%
	Geriatrics^d^	1	0.3%
	Hematology^d^	6	2.0%
	Hepatology^d^	1	0.3%
	Infectious diseases^d^	15	4.9%
	Intensive care medicine^d^	4	1.3%
	Internal medicine^d^	16	5.2%
	Medical oncology^d^	21	6.8%
	Medical ethics^d^	1	0.3%
	Nephrology^d^	8	2.6%
	Neurology^d^	14	4.6%
	Neurosurgery^c^	5	1.6%
	Obstetrics and gynecology^c^	11	3.6%
	Ophthalmology^c^	4	1.3%
	Orthopedics^c^	2	0.7%
	Otorhinolaryngology^c^	4	1.3%
	Pathology^d^	2	0.7%
	Pediatrics^d^	12	3.9%
	Physical medicine and rehabilitation^d^	1	0.3%
	Psychiatry^d^	3	1.0%
	Public health^d^	5	1.6%
	Pulmonology^d^	22	7.2%
	Radiation oncology^d^	7	2.3%
	Rheumatology^d^	11	3.6%
	Surgery, general^c^	7	2.3%
	Surgery, oncologic^c^	8	2.6%
	Surgery, pediatric^c^	1	0.3%
	Surgery, transplant^c^	1	0.3%
	Surgery, vascular^c^	4	1.3%
	Urology^c^	4	1.3%
	Multiple non-surgical specialties	38	12.4%
	Multiple surgical specialties	8	2.6%
	Non-surgical and surgical specialty	2	0.7%
Years of clinical experience as medical doctor^a^	< 5 years	12	3.6%
	5–10 years	30	9.1%
	> 10 years	288	87.3%
Current number of working days in clinical practice per week^b^	0	40	12.1%
	1	24	7.3%
	2	48	14.5%
	3	31	9.4%
	4	22	6.6%
	5	117	35.3%
	6	25	7.6%
	7	17	5.1%

^a^Not filled in by 1 respondent

^b^Not filled in by 7 respondents

^c^Surgical specialty

^d^Non-surgical specialty

## Results

### Characteristics of eligible participants

A total of 393 persons participated. Of these 393 respondents, 37 were excluded because they did not have an MD degree and 25 were excluded because radiology and/or nuclear medicine was their medical specialty. Therefore, 331 clinicians were finally included. Most clinicians were aged 55–64 years (26.9%), were male (74.3%), worked in an academic hospital (81.9%), worked in the USA (19.3%), were a medical specialist (92.7%), had a non-surgical specialty (82.8%), had > 10 years of clinical experience as MD (87.3%), and indicated to work in clinical practice 5 days per week (35.3%) (Table 2). A total of 296 clinicians (89.4%) indicated to request diagnostic radiology examinations in clinical practice, of whom 235 (79.4%) request radiography, 253 (85.5%) request ultrasonography, 270 (91.2%) request CT, and 258 (87.2%) request MRI.

### Value attributed to medical imaging

Clinicians gave a median score of 9 (interquartile range [IQR]: 8–10; range: 1–10) on a 0–10 point scale to the value of medical imaging in improving patient-relevant outcomes. Australia as the continent of work was significantly associated with lower scores (*β* coefficient of − 1.132, 95% confidence interval [CI]: − 2.026 to − 0.238, *p* = 0.013) than Europe as the continent of work.

### Dependency of clinicians on radiologists: radiography

40.6% of clinicians indicated to interpret more than half of radiographs completely by themselves (i.e., without consulting a radiologist or reading the radiology report). In addition, 9.0% reported to never consult a radiologist or read a radiology report for the interpretation of a radiograph (Table 3). Clinicians working in surgical specialties (*β* coefficient of 1.238, 95% CI: 0.602 to 1.875, *p* < 0.001) and clinicians working in Asia (*β* coefficient of 1.304, 95% CI: 0.506 to 2.101, *p* = 0.001) were significantly less dependent on radiologists in interpreting radiographs than those working in non-surgical specialties and those working in Europe.

**Table 3:** Percentages of clinicians (with absolute numbers between parentheses) who indicated which proportion of diagnostic radiology examinations they interpret completely by themselves, without consultation of a radiologist or reading the radiology report

Proportion completely self-interpreted examinations	Radiography^a^	Ultrasonography^a^	CT^b^	MRI^b^
None	26.5% (*n* = 62)	62.7% (*n* = 158)	57.4% (*n* = 152)	67.2% (*n* = 170)
1–25%	23.5% (*n* = 55)	13.5% (*n* = 34)	15.1% (*n* = 40)	15.8% (*n* = 40)
26–50%	9.4% (*n* = 22)	5.2% (*n* = 13)	8.7% (*n* = 23)	7.5% (*n* = 19)
51–75%	9.8% (*n* = 23)	3.6% (*n* = 9)	8.7% (*n* = 23)	5.9% (*n* = 15)
76–99%	21.8% (*n* = 51)	4.0% (*n* = 10)	6.8% (*n* = 18)	2.4% (*n* = 6)
100%	9.0% (*n* = 21)	7.5% (*n* = 19)	3.4% (*n* = 9)	1.2% (*n* = 3)

^a^Not filled in by 1 respondent

^b^Not filled in by 5 respondents

### Dependency of clinicians on radiologists: ultrasonography

15.1% of clinicians indicated to interpret more than half of ultrasonography examinations completely by themselves. In addition, 7.5% reported to interpret ultrasonography always completely by themselves (Table 3). Clinicians working in surgical specialties (*β* coefficient of 0.574, 95% CI: 0.060–1.088, *p* < 0.001) were significantly less dependent on radiologists in interpreting ultrasonography than those working in non-surgical specialties. On the other hand, clinicians working in a non-academic hospital (*β* coefficient of − 1.297, 95% CI: − 2.258 to − 0.337, *p* = 0.008) and clinicians working in North America (*β* coefficient of − 0.618, 95% CI: − 1.115 to − 0.121, *P* = 0.015) were significantly more dependent on radiologists in interpreting ultrasonography than those working in academic hospitals and those working in Europe.

### Dependency of clinicians on radiologists: CT

18.9% of clinicians indicated to interpret more than half of CT scans completely by themselves. In addition, 3.4% reported to interpret CT always completely by themselves (Table 3). Clinicians working in a surgical specialty (*β* coefficient of 0.800, 95% CI: 0.293–1.307, *p* = 0.002) and clinicians working in Asia (*β* coefficient of 0.785, 95% CI: 0.115–1.455, *p* = 0.022) or South America (*β* coefficient of 1.339, 95% CI: 0.158–2.520, *p* = 0.026) were significantly less dependent on radiologists in interpreting CT than those working in non-surgical specialties and those working in Europe.

### Dependency of clinicians on radiologists: MRI

9.5% of clinicians indicated to interpret more than half of MRI scans completely by themselves. In addition, 1.2% reported to interpret MRI always completely by themselves (Table 3). Clinicians working in Asia (*β* coefficient of 0.732, 95% CI: 0.048–1.415, *p* = 0.036) were significantly less dependent on radiologists in interpreting MRI than those working in Europe.

### Projected medical imaging utilization

Medical imaging utilization was expected to increase in the coming 10 years by 289 clinicians (87.3%) and to decrease by 9 clinicians (2.7%), while 33 clinicians (10.0%) remained undecided. None of the clinicians’ characteristics showed any significant association with the projected medical imaging utilization.

### Projected radiologist workforce requirement

The need for diagnostic radiologists in the coming 10 years was expected to increase by 162 clinicians (48.9%), to remain stable by 85 clinicians (25.7%), and to decrease by 47 clinicians (14.2%), while 37 clinicians (11.2%) remained undecided. Clinicians aged > 65 years expected less diagnostic radiologists to be necessary (*β* coefficient of − 0.704, 95% CI: − 1.397 to − 0.011, *p* = 0.046) than clinicians aged 55–64 years.

### Projected impact of AI on the need for diagnostic radiologists

Two hundred clinicians (60.4%) did not expect AI to make diagnostic radiologists redundant in the coming 10 years and 54 clinicians (16.3%) thought the opposite, while 77 clinicians (23.3%) remained undecided. Female clinicians were significantly less likely to expect that AI would make diagnostic radiologists redundant (odds ratio of 0.331, 95% CI: 0.123 to 0.891, *p* = 0.029) than male clinicians.

### Qualitative analysis narrative comments

Most of the 121 clinicians who provided narrative comments at the end of the survey indicated that radiologists will remain necessary in the foreseeable future and that AI may potentially support them in doing their work (particularly for narrow task-specific applications), but that AI is unlikely to replace radiologists (Supplementary Table 1). Many clinicians postulated that AI will be unable to take over human problem solving and to deal with complex or uncommon cases (Supplementary Table 1).

## Discussion

The results of this study indicate that clinicians generally regard medical imaging as very high-value care. Clinicians working in the continent of Australia tended to assign slightly lower grades to the value of medical imaging, but it remains unclear why this was the case.

Our results also indicate that clinicians need radiologists more for cross-sectional imaging than radiography interpretation. In fact, a substantial proportion of radiographs appears to be interpreted completely independently by clinicians without consultation of a radiologist or reading the radiology report. Clinicians working in surgical specialties were significantly less dependent on radiologists in interpreting radiography, ultrasonography, and CT than those working in non-surgical specialties, which may be explained by a greater anatomical knowledge. However, this was not the case for MRI, which is probably due to the more complex physical principles of MRI that need to be understood for image interpretation. Our study also found intercontinental differences in clinicians’ dependency on radiologists in interpreting medical imaging examinations, which may reflect differences in the availability of radiologists and medical imaging skills of clinicians in the various continents. Clinicians working in a non-academic hospital also tended to be more dependent on radiologists for ultrasonography interpretation than those working in an academic hospital. The reason for this observation remains unclear.

Clinicians expected that the use of medical imaging will further increase in the coming 10 years. Almost half of clinicians also expected that more diagnostic radiologists are needed, although those aged > 65 years tended to indicate that less diagnostic radiologists are required. Perhaps retired clinicians are less well informed about the latest developments in imaging technology and applications, which may explain their view on this topic. Most clinicians think that AI will not make diagnostic radiologists redundant in the next 10 years. In fact, most believe AI cannot replace radiologists, and that it can only be used as a support for narrow task-specific applications. Female clinicians were more likely to believe that AI would not replace radiologists, for which we do not have a clear explanation.

Low-value care has been defined as care that confers no benefit or benefit that is disproportionately low compared with its cost [15]. Imaging and other diagnostic studies were described as low-value services in a recent *New England Journal of Medicine* article [16], and it has been estimated that 20–50% of all radiological examinations are of low value [17]. Previous articles have discussed how value may potentially be improved in radiology and how a waste of healthcare resources can be minimized [18, 19]. However, our results indicate that clinicians generally regard medical imaging as crucial for improving patient-relevant outcomes and that their number of medical imaging requests will increase in the future. These findings may perhaps convince policymakers that it is necessary to invest in medical imaging.

Previous studies have shown that around 10–20% of radiology reports are not read by clinicians [20, 21]. Previous work also indicated that radiography reports are less frequently read than cross-sectional imaging reports [20]. In a study among 81 orthopedic surgeons working in hospitals in German-speaking regions of Europe, radiography, CT, and MRI reports were routinely viewed by 43%, 67% and 86%, and were never viewed by 20%, 4%, and 0%, respectively [22]. In another study among 200 orthopedic surgeons in Australia and New Zealand, reports for radiography, ultrasonography CT, and MRI were read by 10%, 74%, 35%, and 92%, respectively [23]. The results of these previous studies resonate with ours, and may trigger policymakers to reconsider if radiologists should report all diagnostic examinations (particularly radiographs) or limit their time and energy to examinations for which their assistance is really needed (particularly cross-sectional imaging). Implementing such a reform in clinical routine could save valuable healthcare resources and increase the value of diagnostic radiologists.

In the past, an increase in the demand for medical imaging services would simply imply that the number of diagnostic radiologists also had to increase to compensate for the increased workload [24]. However, AI is considered as a disruptive technology whose impact on the required workforce of diagnostic radiologists still remains unclear [25]. In a study from 2018, 56 surgeons in Switzerland were asked their opinion about the role of radiologists and the future of radiology [26]. The surgeons in that survey were somewhat equivocal as to whether or not the required workforce of diagnostic radiologists will change and how it will be affected by AI [26]. In the present study, however, clinicians generally tended to predict a need for more radiologists in the coming 10 years and did not expect AI to replace radiologists. This difference may be explained by the fact that the present study included a larger and more representative group of clinicians, including those with a non-surgical specialty. In addition, clinicians’ knowledge about the possibilities and limitations of AI may have evolved in the past few years. These projected workforce requirements may be informative to policymakers who decide about the number of radiology residents that should be trained and how many financial resources should be allocated to employ (new) radiologists.

The present study had some limitations. First, this study was limited to corresponding authors who published in the *New England Journal of Medicine* or the *Lancet*, and predominantly included clinicians working in an academic hospital. Second, this study provided an understanding of clinicians’ current view on some important aspects of diagnostic radiology, but this may change in the near future. Third, although our study showed that clinicians interpret a considerable proportion of imaging examinations completely by themselves (i.e., without consulting a radiologist or reading the radiology report), it remains to be further investigated to which specific clinical settings this practice applies. Fourth, clinicians were asked whether they requested plain radiography, ultrasonography, CT, and MRI examinations in their practice and which proportion of these examinations were completely interpreted by themselves. However, plain radiography, ultrasonography, CT, and MRI are imaging modalities and not specific types of images. Breast imaging, for example, was not addressed. Furthermore, this study did not specifically investigate the clinicians’ view on the value and future of nuclear medicine imaging. These topics should be investigated by future studies. Fifth, the survey was not formally validated prior to using for the present study, and the qualitative analysis was not done using a formal model and process.

In conclusion, clinicians who published in the *New England Journal of Medicine* or the *Lancet* attribute high value to medical imaging. They generally need radiologists for cross-sectional imaging interpretation, but for a considerable proportion of radiographs, their service is not required. Most expect medical imaging utilization and the need for diagnostic radiologists to increase in the foreseeable future, and do not expect AI to make radiologists redundant.

### Supplementary information

Below is the link to the electronic supplementary material.

Supplementary file1 (PDF 210 KB)

## Abbreviations

AI: Artificial intelligence
CI: Confidence interval
IQR: Interquartile range
MD: Medical doctor

## Declarations

### Guarantor

The scientific guarantor of this publication is Thomas C. Kwee.

### Statistics and biometry

No complex statistical methods were necessary for this paper.

### Study subjects or cohorts overlap

None of the study subjects has been reported previously.

### Methodology

- not applicable
- experimental
- performed at one institution

## References

[1] Levine D, Kressel HY (2014). Exploring the evolution of imaging. Radiology.

[2] Smith-Bindman R, Kwan ML, Marlow EC (2019). Trends in use of medical imaging in US health care systems and in Ontario, Canada, 2000–2016. JAMA.

[3] Balasubramaniam R, Subesinghe M, Smith JT (2015). The proliferation of multidisciplinary team meetings (MDTMs): how can radiology departments continue to support them all?. Eur Radiol.

[4] Mirnezami R, Nicholson J, Darzi A (2012). Preparing for precision medicine. N Engl J Med.

[5] Porter ME (2009). A strategy for health care reform–toward a value-based system. N Engl J Med.

[6] Herold CJ, Lewin JS, Wibmer AG (2016). Imaging in the age of precision medicine: summary of the proceedings of the 10th biannual symposium of the International Society for Strategic Studies in Radiology. Radiology.

[7] Brady AP, Bello JA, Derchi LE (2021). Radiology in the era of value-based healthcare: a multi society expert statement from the ACR, CAR, ESR, IS3R, RANZCR, and RSNA. J Am Coll Radiol.

[8] Dieleman JL, Cao J, Chapin A (2020). US health care spending by payer and health condition, 1996–2016. JAMA.

[9] Goryakin Y, Thiébaut SP, Cortaredona S (2020). Assessing the future medical cost burden for the European health systems under alternative exposure-to-risks scenarios. PLoS One.

[10] Bruls RJM, Kwee RM (2020). Workload for radiologists during on-call hours: dramatic increase in the past 15 years. Insights Imaging.

[11] Hosny A, Parmar C, Quackenbush J, Schwartz LH, Aerts HJWL (2018). Artificial intelligence in radiology. Nat Rev Cancer.

[12] Alexander A, Jiang A, Ferreira C, Zurkiya D (2020). An intelligent future for medical imaging: a market outlook on artificial intelligence for medical imaging. J Am Coll Radiol.

[13] Alvarado R (2022). Should we replace radiologists with deep learning? Pigeons, error and trust in medical AI. Bioethics.

[14] Bonekamp D, Schlemmer HP (2022). Artificial intelligence (AI) in radiology?: do we need as many radiologists in the future?. Urologe A.

[15] Scott IA, Duckett SJ (2015). In search of professional consensus in defining and reducing low-value care. Med J Aust.

[16] Zaki MM, Jena AB, Chandra A (2021). Supporting value-based health care - aligning financial and legal accountability. N Engl J Med.

[17] Kjelle E, Andersen ER, Soril LJJ, van Bodegom-Vos L, Hofmann BM (2021). Interventions to reduce low-value imaging - a systematic review of interventions and outcomes. BMC Health Serv Res.

[18] Bradley D, Bradley KE (2014). The value of diagnostic medical imaging. N C Med J.

[19] Larson DB, Durand DJ, Siegal DS (2017). Understanding and applying the concept of value creation in radiology. J Am Coll Radiol.

[20] Hurlen P, Østbye T, Borthne A, Dahl FA, Gulbrandsen P (2009). Do clinicians read our reports? Integrating the radiology information system with the electronic patient record: experiences from the first 2 years. Eur Radiol.

[21] Reda AS, Hashem DA, Khashoggi K, Abukhodair F (2020). Clinicians’ behavior toward radiology reports: a cross-sectional study. Cureus.

[22] Donners R, Gutzeit A, Gehweiler JE, Manneck S, Kovacs BK, Harder D (2021) Orthopaedic surgeons do not consult radiology reports. Fact or fiction? Eur J Radiol 142: 109870 10.1016/j.ejrad.2021.109870 34304032

[23] Kruger P, Lynskey S, Sutherland A (2019). Are orthopaedic surgeons reading radiology reports? A Trans-Tasman Survey. J Med Imaging Radiat Oncol.

[24] Rosenquist CJ (1995). How many radiologists will be needed in the years 2000 and 2010? Projections based on estimates of future supply and demand. AJR Am J Roentgenol.

[25] Mazurowski MA (2019). Artificial intelligence may cause a significant disruption to the radiology workforce. J Am Coll Radiol.

[26] Van Hoek J, Huber A, Leichtle A (2019). A survey on the future of radiology among radiologists, medical students and surgeons: students and surgeons tend to be more skeptical about artificial intelligence and radiologists may fear that other disciplines take over. Eur J Radiol.

