# Supplementary material for: Diagnostic radiology and its future: what do clinicians need and think?
Source: Eur Radiol. 2023 Jul 12;33(12):9401–10. doi: 10.1007/s00330-023-09897-2 (PMC10667510; doi:10.1007/s00330-023-09897-2)
Supplement: Supplementary file 1 — Supplementary file1 (PDF 210 KB) [file 330_2023_9897_MOESM1_ESM.pdf]

Diagnostic radiology and its future: what do clinicians need and think?

Electronic Supplementary Material

Supplementary Table 1. Narrative comments provided by 121 clinicians at the end of the survey.

No.	Comments
1	One of the benefits of removing radiologists from interpreting most scans is that it will prevent the relentless increase in costly imaging. Basically, in a private or fee for service system they always suggest more follow up imaging and ancillary tests than is necessary. They are NEVER interested in cost effectiveness.
2	You need the radiologist to put the AI diagnosis into context. Therefor radiologists in the future will need more clinical experience.
3	While AI will impact interpretation of standard radiologic studies, I think new modalities (PET and new things to come) will add greater need for human expertise given the lack of data supporting AI learning.
4	We'll have to wait for the validation studies on AI.
5	We will incorporate AI but it will not replace radiologists. We need to be able to discuss cases with radiologists and discuss the best management for our patients.
6	We still need radiologists.
7	We need more specialized radiologists (e.g. radiologists with a dedicated focus in interventional cardiology), or specialists with radiological competence (such as interventional cardiologists who have radiological expertise).
8	We have been working on a project using AI to interpret pulmonary US. The work shows the complexity of the challenge and makes me extremely sceptical that a computer algorithm will be able to take this task on. I think the likelihood is much greater that AI could be used for certain tasks that are very specific. Eg., is there a

	breast mass on this mammogram? I have less confidence that AI can answer an open-ended question, such as 'what is the likely diagnosis in this patient given the following clinical data and the current radiologic study?"
9	We can’t deny that AI will be the future bcos human minds curiosity and exploration. Thus, advancement in technology is inevitable. Its up to us to use them wisely.
10	Using BoneExpert for bone ages, which is fantastic.
11	Ultrasound images are hard to review, and it is much easier performing the scan and interpreting the result at the same time. This could not be done with AI.
12	Think that AI will aid radiology interpretation but will not replace radiologists.
13	They will do different jobs; helping to decide which diagnostic modality should be used. participate in multidisciplinary meetings. If they are interventionalists they have to learn to behave as doctors (talk to patients, record keeping, post-intervention care, etc.)
14	They will be useful but always room for clinical judgement.
15	there continues to be a false notion that AI will replace human problem solving. AI is merely a tool. Did we think that a electric saw would replace humans cutting wood? But it made it easier and faster to complete the work. AI is a tool that will make it easier and faster to complete the work. That's it, that's all.
16	There are some things on radiological scans that only an experienced eye can detect and interpret correctly. AI is amazing but there still has to be a human eye to check things otherwise there is a risk of errors.
17	There are problems of responsibility which cannot be taken by a machine only.

18	The standard registration of all plain radiographs might become redundant, ultrasound might be replaced a bit by POCUS, but we definitely need radiologists for the (many) situations that we encounter a uncommon disease or image.
19	The problem with artificial intelligence is that it won’t be able to decide about difficult cases. In that case human judgement will be required. However, if radiologists are not seeing enough scans, they will get deskilled and not be able to spot the rarities.
20	the last two questions would need a more complex answer. I think that the radiologists will still be needed but probably in a different way.
21	The imaging field together with site specific interventions will be a major therapeutic area to advance medicine.
22	The field of child abuse has very nuanced findings that require a combination of science, experience, and "the art of medicine". This cannot be replaced by artificial intelligence.
23	The clinical setting is still critical for interpretation.
24	The artificial intelligence is an evolving technology that definitely will help decrease errors in diagnosis but diagnostic radiologist input is very important particularly in emergencies where the medical history may impact the diagnosis.
25	The answer to the last question is 'No' only because the time mentioned is 10 years. I believe it would happen to a large extent but over a longer time horizon.
26	Supplement, not replace.
27	Some medical plain radiology tests will be reported by machines but many other scans need radiologists to be developed. Humans will always be the validators of machine reports. New radiologic technics based on IA may be developed in future thanks to development of this field.

28	Role of radiologist will change into correcting ai diagnoses and connecting findings to clinical indication/question. they will not disappear but adapt.
29	Radiology will evolve with AI and other innovations. But they will remain necessary in most countries (I anticipate).
30	Radiologists provide unique insight taking the clinical picture into account, which helps diagnosis and management.
31	Radiologists are invaluable in their interpretation, marrying what is seen in an image to the Clinical status, history and condition of the patient. For this they utilise their clinical acumen. We are some way away from an artificial intelligence that has some experience in managing patients' medical conditions.
32	Radiologists are essential to read and interpret images ideally enhanced by AI.
33	Radiologists always needed, although the number of radiologists may decrease in time, as the role of AI will increase.
34	Radiologists also have a huge role in interventional procedures, catheter directed thrombolysis, deep tissue biopsy etc.
35	Radiologists are and will remain invaluable. AI cannot replace a human brain and experience for complex diagnosis.
36	Quite often an experienced radiologist is helpful in defining complex cases.
37	Q18 is poorly worded and somewhat biased around the word "redundant". Redundant is too broad; it is not the case *all* diagnostic radiologists will be "redundant", but it is certainly possible AI augmented processes will automate some portion of diagnostic radiology workflow and make some diagnostic radiologist tasks – and thereby some diagnostic radiologists – redundant. So, the answer is "yes", but the answers don't allow for enough nuance to accurately describe what "redundant" means to the person answering the question.

38	Q13 and Q14. My answer was none, which might incorrectly imply that I am very dependent on radiologists. I always interpret the images myself but always read the report in case I have missed something.
39	Probably a complementary role to help ex: find nodules on thoracoic ct scan but radiologists will still be needed for diagnosis and interventional procedures:
40	Probably > 10 years to redundancy:
41	PET-CT will be even more important in the future.<br>I am not sure I will trust in a machine for interpretation in the future 10 years.
42	People will be increasingly aware of the risks of radiation and radiologists will have to use methods other than X-rays, for which there is currently no AI support.<br>https://danielcorcos.substack.com/p/bca
43	Patient care is a team approach. That’s why when a critical juncture comes in a patient’ s care, that a collaborative effort in review films is necessary. As an oncologist i can provide the history of the patient, which is necessary to understand why we are doing a procedure. I think that AI will help but it does not replace human interaction:
44	OPHTALMO Ultrassonigrafists do all the US:
45	Not redundant, but with increasing workload AI may alleviate the burden of work.
46	No doubt, artificial intelligence will make the first diagnosis but then it will be the doctor responsible for the patient who will make the final interpretation.
47	New imaging techniques (driven by imaging companies and patient wishes) and image-guided operations will warrant the ongoing need for diagnostic radiologists<br>AI is as good as the reliability/quality and number of its input variables and needs to be validated by diagnostic radiologists.

	The value of imaging for patients' outcomes depends on the adequacy of ordering clinical relevant imaging.
48	Need radiologists to discuss findings in particular contexts and based on their experience. AI might be able to take some of the superficial load off, but the complex cases will continue to require radiologists. data driven medicine is all very well but you can't beat experience and contextual assessments.
49	My expertise is in tuberculosis control worldwide and diagnostic research. I foresee a steep increase in the importance and utilization of chest X-rays for TB screening, especially in low and middle income countries that have high incidences of TB. This will possible due to two developments: low-radiation low-cost mobile digital X-ray, and computer-assisted diagnosis. The latter will obviate the need for radiologists to read these (screening) X-rays.
50	Modern imaging should be the first and or second measure to get rapid information regarding status of any disease, including infection and tumor status<br>save a lot of time and accelerates all decisions dramatically.<br>should be automated as much as possible probably including so called AI.
51	Medical specialists are best placed to interpret the radiographic/MRI findings in the clinical context. Therefore radiologists should stay away from making clinical diagnoses in rheumatology. They can only provide a clear-cut description of what they see (sometimes on top of what was asked).
52	Machine learning does not know medical history.
53	It is impossible to make a clear assumption how much AI will replace diagnostic radiologists. It will certainly change the work of the diagnostic radiologist.

54	It is a difference in emergency situations, then of course I can evaluate an emergency CT or MRI myself. Regarding AI and different software programs, we should look upon them as a help, not as an instead of.
55	Interventional radiology will remain important.
56	Interpretation of AI results will be needed in the future - throughout medicine.
57	Interpretation critical.
58	Interaction between clinician and radiologist, with discussion of clinical features, essential for correct interpretation.
59	In my specialty I don’t think diagnostic imaging will increase but I also don’t think it will decrease and I do not think AI can replace the diagnostic acumen that I rely upon for rare bone diseases.
60	In low-income settings AI offers some opportunities, but this is mainly because there are so few radiologist. While AI may be useful in doing pre-report or reports of completely normal imaging, I do believe that radiologists or experts are needed to fine tune and interpret.
61	In future, specialist training / skill set of radiologists may need to be adapted to more frequent usage and understanding pitfalls of integrating AI in diagnostic work-ups.
62	Improvements in imaging technology have increased the diagnostic value of imaging, and vastly increased the complexity of interpretation. Good radiologists do not just interpret images, they provide clinical context. The best radiologists are not just expert at image recognition, they are expert clinicians as well.
63	Imaging physicians should be replaced.
64	If radiologists do not build strong technical expertise and fruitful relationships with specialists, they will be replaced by AI both in private and academic practice, or will be outsourced to Third World countries.

65	If AI is used to make a diagnosis, with clinical consequences, then oversight and ultimate responsibility should remain with a clinical specialist.
66	I think there is much to gain and many AI algorithms already outperform radiologist. So let's embrace them and let the radiologist spent more time on truly difficult case and MDT's.
67	I think the work I do in the UK is very different form the work I do in Zambia. In the UK I use the radiology report. In Zambia that doesn't exist so AI could have a larger role.
68	I think that imaging will increase in frequency, and that radiologists will increasingly utilise AI to assist them. But I don't see AI replacing diagnostic radiologists, certainly not within 10 years.
69	I think artificial intelligence will be an aid to radiologist and make them more efficient. It may reduce their need but to me not likely to replace them.
70	I think AI will replace radiologists in some settings (e.g. screening) and in some diagnostic radiology. However, there will still be a role for human radiologists.
71	I think AI will no doubt play a major role in routine interpretations, but there will be no substitute for the personal interaction and case discussion with an experienced radiologist.
72	I think AI will make a substantial difference to diagnostic imaging but I do not think this will make radiologists redundant.
73	I think AI may mean that we need fewer diagnostic radiologists for some tasks where there is evidence that AI performs as well or better than human interpretation or where AI can triage images to allow radiologists to focus attention on abnormal images.

74	I suspect that AI-assisted radiology will become more prominent but don't think that AI will completely replace diagnostic radiologists.
75	I see ai and machine learning as supplementing not replacing.
76	I presume you do mean radiologists, not radiographers.<br>Key emerging discipline for the future.
77	I perform all my own TEE exams and interpret myself for Cardiac Surgeries.
78	I often review the MRI and CT imaging for findings of the inner ear related to neurotology and skull base. I rely on the radiologist interpretation, but there is varying levels of expertise among radiology for these types of scans, so I sometimes recognize things they didn't see and vice versa. The radiologist has such a broad training that they can pick up also incidental things that I would never have seen. Perhaps this can be replaced by AI, as we all can, but I suspect it's much more likely that their practice will be augmented by AI, rather than replaced.
79	I have had very good experience with interventional radiology, and I think the role of this approach will increase.
80	I don’t think that AI can take place of medical doctors.
81	I believe we will end up with a combination of AI and radiologists, each enhancing the other. However, overall, because of incorporating AI, we will need less radiologists in total.
82	I believe that AI will lead to a dramatic change in how doctors work, and this will happen faster for radiologists and pathologists. It will change the way we work, but I do not think that we will need less doctors.
83	I am convinced it will take much longer for AI to make radiologists redundant, if that ever will happen.

84	I am confident that AI will change all of medicine including Radiology but not make physicians irrelevant. But the roles of physicians will change and the nature of practices will change too. I welcome the changes. This is as it has been since the beginning of "the practice of medicine and will be forever more.
85	I always read the radiologists report but do not necessarily agree with them, especially with chest x-rays, an area of my special expertise. In the US, radiologists are grossly overpaid, especially since they lack the demands of direct patient responsibility and much of the routine work they do could be replaced by AI. Is there some reason you queried physicians in Saudi Arabia and Egypt but not in Israel? Good luck with your work.
86	Good radiology is essential. what we see is the community radiology standards are dropping and we are more than ever reliant on strong academic radiology. AI might help some niche areas but it won't synthesise information on complex disease presentations.
87	Given the lower emphasis on anatomy in the medical courses, few new graduates can feel confident about knowing variations in anatomy and being able to interpret pathology vs variations.
88	For some simple images, the answer is yes.
89	For screening and routine, AI will make radiologist redundant but never for complicated cases.
90	for Q13, I do look 1st to the CT Scan for my own evaluation with the information already given by the patient before to always look back to the radiologist report<br>for this survey : question is not so much the number of radiologist but for what to do. for standard imaging the need will be far less due to AI. but if we consider that AI + "omics" will take more part there will be more need for expert radiologist to

	understand and to improve the quality of care at a similar level as pathologist, molecular pathologist are going to be<br>the question will be how the patients in the community far from expert centers will have access to that. so probably "general" radiologist to coordinate at the local level:
91	Diagnostic radiologist will be helpful only for some imaging reviews at MDT or pre-operatively.
92	Clinical discussion with an experienced and well trained radiologist, can be very enriching to understand the complexity of some complex clinical situations.
93	Assuming that interventional radiology will continue to increase.<br>I imagine that radiology reporting will increasingly be centralised and delegated to non-medical stand.
94	As in all fields, the work will change but the need for good imaging can only increase as is key to clinical decision making.
95	As a specialist in uncommon CV conditions, I interpret all of my own imaging studies and often bring attention to the radiologist things they either missed or things they say in the report that indicates they do not understand the disease process. Plus, I direct a vascular diagnostic laboratory that performs more than 10,000 studies per year.
96	Artificial intelligence should not replace radiologist. It should be a complementary tool.
97	AI/ML can make radiologists' work more efficient (and thus reduce workload), but on the other hand expanding indications and an ageing population will likely increase workload. So on balance, if we arrange things efficiently and make use of AI, I think the total number of radiologists needed will remain approximately the same over the coming 20 years. Content of the work will change.

98	AI will support some decisions, help for screening but there other areas, like functional imaging and interventional radiology, as examples, that will need more radiology human resources.
99	AI will not replace humans for the foreseeable future.
100	AI will increase very much the accuracy, discriminative and diagnostic power of medical imaging. In the very long term it might make the work of the radiologist redundant. I think we will not see that in our generation, so imaging specialists will be required. The role of the radiologist will evolve as it has been evolving. While general radiologists will remain being important, the within-specialty super-specialization in some fields has created "medical imaging specialists" within some specialties (i.e. cardiology or fetal medicine for instance), and this will continue to evolve further into flexible and interdisciplinary profiles.
101	AI will improve work rate but not substitute for radiology expertise.
102	AI will help radiologist but not replace them any more than computers have displaced mathematicians.
103	AI will be able to evaluate straightforward examinations and help with FU after EVAR, FEVAR ... to detect problems better than humans.<br>But it will also identify these investigations that require human input.
104	AI will assist the Human readers.
105	AI will allow expansion & efficiency of current tests (automated pre-detection/segmentation) and expand indications (MR fingerprinting, image-guided biopsy tracking) but number of radiologists will be stable.
106	AI will affect the future of radiology - more radiological intervention will be needed due to increased number of procedures required for incidental findings and repeat scans etc.

107	AI will address specific issues, like measuring lesions on serial scans, speeding up workflows, possibly reducing workload in screening exams, and facilitating image review. It will not replace wide medical or imaging knowledge for a very long time, possibly never. I already use a lot of AI tools and they just add to the workload, frequently miss or misinterpret the feature of interest, over complicate an already complicated job and slow everything down. I'm sure they can be made to work better eventually.
108	AI remains unproven.
109	AI PROS CONS
110	AI may increase the efficiency of radiologists.
111	AI is overvalued.
112	AI is not likely to help individuals in place of diagnostic radiology. I shudder to think of not having their input.
113	AI is likely to automate some simple reporting (e.g. line, tube placement) but it's unlikely we'll ever allow human-out-of-loop reporting in health - expert input will always be needed, especially for complex and nuanced reporting.
114	AI is far fetched as replacement for radiologist. Even many human radiologists fail to report the precise pathology we request to delineate.
115	Ai cannot replace human intellect. Clinical correlation is always required.
116	AI can only screen, then a radiologist need to confirm.
117	AI adds quality but will not be able to take away the complexity of radiological interpretation and in fact will add some complexity (interpretation of results, pitfalls). It may help reduce the increase in number of radiologists needed in the ever growing field of medicine.

118	Absolutely absurd question.<br>Of course we will need people with radiological expertise.
119	1. Interpretation of imaging will be done best by AI,<br>2. 10 years is too short time,<br>3. operator-dependent techniques may still benefit from radiologist,<br>4. I assume that interventional radiologists, even for diagnostic purposes, are not being discussed here.
120	The experience of a radiologist in a given field is fundamental. IA will help to pick general anomalies.
121	AI will replace some basic interpretations at present performed by radiologist.<br>For complex cases like follow-up of Crohn's disease AI will be of added value. The interdisciplinary discussion will however still be necessary.
